# An intervention study of poly-victimization among rural left-behind children based on the theoretical framework of planned behavior

**DOI:** 10.1186/s13034-024-00812-1

**Published:** 2024-09-12

**Authors:** Yandong Luo, Jiajun Zhou, Pan Wen, Ping Chang, Zicheng Cao, Liping Li

**Affiliations:** 1 School of Public Health, Shantou University, Shantou, 515041 P.R. China; 2 Injury Prevention Research Center, Shantou University Medical College, Shantou, 515041 China

**Keywords:** Left-behind children, Theory of planned behavior, Intervention research

## Abstract

**Background:**

Poly-victimization (PV) not only threatens physical and mental health but also causes a range of social problems. Left-behind children in rural areas are more likely to experience PV problems. However, there have been fewer studies on PV among rural children, and even fewer intervention studies.

**Objective:**

The difference-in-differences method was employed to analyze the impact of intervention measures, based on the theory of planned behavior, on PV among left-behind children in rural areas.

**Methods:**

The study subjects were left-behind children from six middle schools in two cities in southern China, who completed the baseline survey from 2020 to 2021. They were divided into a control group and an intervention group, each consisting of 228 cases, based on their schools. Before and after the intervention, the Self-made victimization-related knowledge, attitude, and practice questionnaire, Poly-victimization scale, and Middle school students’ coping style scale were used to evaluate the victimization-related KAP(knowledge, attitude, and practice), victimization occurrence, and coping styles of left-behind children, respectively. Stata 15.0 was used to establish a difference-in-differences regression model to analyze the impact of the intervention measures on poly-victimization and coping styles.

**Results:**

Mixed Anova revealed that after the intervention, the KAP scores of the intervention group were significantly higher than those of the control group (*p* < 0.05). After the intervention, the incidence of child victimization in the intervention group dropped to 9.60% (*n* = 22), lower than in the baseline survey, with a statistically significant difference (*p* < 0.01). The incidence of PV among children in the intervention group was lower than that in the control group, with the difference being statistically significant (*p* < 0.01). The net reduction in the incidence of PV among children was 21.20%. After the intervention, the protection rate for preventing PV among children was 73.33%, and the effect index was 3.75. The intervention improved children’s coping styles, problem-solving, and help-seeking, while reducing negative coping styles such as avoidance and venting, with the differences being statistically significant (*p* < 0.05).

**Conclusion:**

Intervention measures based on the theory of planned behavior reduce the occurrence of PV among left-behind children, and the intervention effects on different types of victimization are also different.

## Introduction

Left-behind children in rural areas are a unique group among children in China. Rural left-behind children are defined as children under the age of 18 who reside in rural areas and have one or both parents working as migrant laborers in urban areas [1]. These children are typically left in the care of relatives, such as grandparents, or other guardians. The phenomenon of left-behind children is prevalent in China due to the large-scale internal migration driven by economic opportunities in urban areas. This prolonged separation from their primary caregivers often leads to emotional and social challenges [2]. As of the latest data, more than 33% of children (6.97 million) residing in rural China are left-behind children [3]. This phenomenon is not exclusive to China, similar patterns are observed globally wherever significant rural-to-urban or international labor migration occurs. For instance, substantial numbers of left-behind children are also reported in countries such as India [4], Philippines (27%,8 million) [5], and Thailand (6.6%, 3 million) [6], facing comparable social and emotional challenges.

These children are more vulnerable to victimization due to the lack of parental supervision and support, making them a critical group for study in the context of poly-victimization (PV). Research has shown that this group has a higher probability of experiencing PV problems [7]. PV refers to children experiencing multiple types of harm within the past year, including physical victimization, property victimization, child abuse, peer victimization, sexual victimization, witnessing/indirect victimization, and other forms of victimization [8]. PV not only threatens physical and mental health but also causes a range of social problems such as suicide attempts, post-traumatic stress disorder (PTSD), depressive symptoms, and violent behavior [9, 10]. It can also lead to practical problems in children, such as poor school performance, alcohol abuse, involvement in crime, and revictimization [11, 12]. According to a survey conducted in Sichuan Province, China [13], the PV incidence rate among the general child population was found to be 28.3%. In comparison, left-behind children whose parents have migrated for work exhibited a PV incidence of 28.5%. Notably, left-behind children experiencing parental separation or divorce had a significantly higher PV incidence rate of 39.1%. These statistics highlight the elevated risk of victimization among left-behind children, underscoring the importance of targeted interventions to address their unique challenges. Currently, many studies in China have focused on individual types of victimization among rural children [14–16], but there have been fewer studies on PV in this population, and even fewer intervention studies.

Children use different coping styles when they experience PV as a stressful event. Coping style refers to the cognitive and practical strategies that individuals adopt in the face of frustration and stress, also known as coping mechanisms [17]. Coping styles can significantly influence how children deal with PV, affecting both their immediate reactions and long-term resilience [18]. An individual’s coping style influences the nature and intensity of the stress response and moderates the relationship between stress and its outcomes. The Theory of Planned Behavior (TPB) [19] is a practical decision-making model proposed by Icek Ajzen. It is mainly used to predict and understand human behavior, with a core intervention strategy focused on changing practical intention, which is jointly determined by attitude, subjective norm, and perceived practical control. The theory has been widely applied to many aspects of human life, such as fitness and exercise behavior, healthcare behavior, social learning behavior, and more [20, 21]. Meanwhile, the Theory of Planned Behavior has been successfully applied in various interventions aimed at children and adolescents, demonstrating its relevance and effectiveness in this demographic [22, 23]. Several studies have shown that TPB has been used to investigate or predict children’s diets and undesirable behaviors or encounters, among other things. For example, one study used two theories, including the TPB, to predict smoking among Chinese adolescents and found that the TPB was superior [24]. And another Indian study explored the role of Theory of Planned Behavior in predicting areca nut use among adolescents [25]. Therefore, the coping styles of left-behind children exposed to PV can be regulated and improved by applying this theory, focusing on intervention in attitudes, subjective norms, and perceived practical control.

A baseline survey conducted as part of this study in 2020 established the initial prevalence of PV among left-behind children at 23%(626/2722). This data underscores the critical need for targeted interventions to address the high vulnerability of this group. In response, our study designed and implemented an intervention strategy based on the Theory of Planned Behavior (TPB), aimed specifically at mitigating these risks. The baseline study and its results are an integral part of the sample size calculations for subsequent randomized controlled trials and the analysis of intervention effects in this paper.

The difference-in-differences (DID) model [26] was used to assess the impact of the intervention on the knowledge, attitudes, and practices of left-behind children and the incidence of PV, thereby comprehensively evaluating the effectiveness of the intervention. The Difference-in-Differences (DID) model is a quasi-experimental research design that helps estimate the causal effect of a treatment or intervention. It does so by comparing the changes in outcomes over time between a treatment group (which receives the intervention) and a control group (which does not). This method helps to account for time-invariant differences between the groups and for trends that would affect both groups similarly, thus isolating the effect of the intervention. DID allows us to make robust causal inferences about the impact of our intervention by comparing the pre- and post-intervention outcomes between the intervention group and the control group. Moreover, the DID model is well-suited for this repeated measures data of victimization-related outcomes before and after the intervention. This allows us to control for baseline differences and observe changes over time, providing a clearer picture of the intervention’s effectiveness. DID has been widely used in research on adolescents. In a survey of repeated measures of adolescent risk behavior across 41 continents in the United States, the study used the DID to compare changes in past-year physical TDV (Teen dating violence) in states that enacted TDV laws compared to states with no required laws [27]. In another study of pedestrian injuries in U.S. schoolchildren estimated difference-in-differences in injury risk between census tracts with and without intervention following the changepoint [28].

The purpose of this study was to design and implement a TPB-based PV intervention strategy for left-behind children and to explore its effectiveness in addressing the PV challenges faced by these children, and specifically whether: [1] the TPB-based intervention reduce the incidence of PV among left-behind children [2]. Whether children’s coping styles improved after the intervention, particularly their ability to manage their emotions and communicate their thoughts and feelings to others [3]. Evaluate the effects of the intervention on enhancing children’s Knowledge, Attitude, and Practice (KAP) related to personal safety, peer interactions and so on. Through a community intervention trial, tis study evaluated the effectiveness of these interventions to provide a scientific basis for developing victimization intervention programs for left-behind children in other regions.

## Methods

### Participants

A survey was conducted in six middle schools in two cities in southern China (Shantou and Jieyang) between 2020 and 2021 to establish a baseline. Left-behind children from these schools were randomly assigned to intervention and control groups. Eligible participants were students in the first and second grades of junior high school who were in good health, had typical physical mobility, and were willing to undergo the intervention. The intervention phase was carried out between 2021 and 2022. Inclusion Criteria: [1] the first or second grades of junior high school [2]. Identified as left-behind (having one or both parents working away from home). Exclusion Criteria: [1] Those with significant cognitive or physical disabilities that could interfere with participation in the intervention or assessment procedures, as reported by school administrators or guardians. We sampled 460 students, and based on the above inclusion and exclusion criteria we ended up with 456 students who were randomly assigned to the intervention and control groups (*n* = 228). As shown in Table 1, the mean and standard deviation of children’s ages in the intervention and control groups were 13.28 ± 0.232 and 13.66 ± 0.342, respectively.

### Sample size

Based on the findings of the baseline survey, the initial prevalence of PV among left-behind children was recorded at 23.00%, denoted as P_max = 0.23. Following the implementation of the proposed intervention, the prevalence of PV decreased by 10.00–13.00%, denoted as P_min = 0.13. Utilizing significance levels of α = 0.05 and β = 0.20, with a factor k = 2 representing the number of groups, a critical value of λ = 7.85 was obtained from the relevant table of values. Consequently, a minimum sample size of *n* = 228 was determined, necessitating at least 228 participants in both the intervention and control groups. The formula for this calculation is provided below:

1 $$n = \frac{\lambda }{{2\left[ {\sin ^{{ - 1}} \left( {p_{{\max }} } \right)^{{1/2}} - \sin ^{{ - 1}} \left( {p_{{\min }} } \right)^{{1/2}} } \right]^{2} }}$$

### Procedure

This study selected six middle schools in two cities in southern China as research sites to conduct an intervention study and measure the changes in the knowledge, belief, and behavior levels, as well as the incidence of PV among left-behind children, before and after the intervention.

Research in behavioral psychology suggests that longer interventions are more effective in instilling sustainable changes in behavior and attitudes [29, 30]. Twelve months allow for the gradual assimilation and reinforcement of new behaviors and concepts, essential in changing established habits and norms. And this schedule also aligns with academic calendars, facilitating a structured approach to implementation within school settings, ensuring that interventions do not overwhelm participants while providing regular touchpoints for reinforcement and assessment of progress. Meanwhile, the number of interventions was 12 a year rather than more, taking into account the possibility of students changing schools and the lost visits, as well as the fact that the poly-victimization questionnaire was a survey of victimization sustained in the past year [8].

The study began in September 2021 and continued through August 2022, with thematically relevant content interventions each month for the intervention group and general health and safety education for the control group, for a total of 12 interventions for each group. And a comprehensive assessment was conducted before and after the intervention to assess changes in the incidence of poly-victimization in children as well as changes in participants’ knowledge, beliefs, and behaviors.

### Intervention group

The intervention themes, grounded in the Theory of Planned Behavior [31], aim to modify attitudes, subjective norms, and perceived behavioral control regarding poly-victimization. Themes like Types of PV, School Bullying, and Sexual Safety educate on risks and prevention, aiming to change attitudes and enhance behavioral control [32]. Topics such as Interpersonal Communication and Non-violent Communication foster supportive social norms and improve skills in managing stress and emotional reactions, crucial for preventing poly-victimization [33].

Practical attitudes are raised by explaining to students the types of PV, its characteristics, forms of occurrence, and associated hazards, to raise awareness among left-behind children about PV. Subjective norms primarily involve fostering positive external environments, engaging in interventions with teachers to enhance their understanding of PV, and equipping them with the skills to identify individuals at risk and prevent such incidents.

Improvement in perceived practical control was achieved by having students record instances of victimization and their responses. Finally, case studies and discussions were conducted to promote changes in the practical intentions of left-behind children towards beneficial actions.

Changes in Knowledge (K): Knowledge enhancement was achieved through educational sessions that provided information about PV, its signs, consequences, and prevention strategies [9]. These sessions were interactive, using multimedia presentations, discussions, and Q&A sessions to ensure participants comprehended and retained the information. Pre- and post-tests were used to measure changes in knowledge levels.

Changes in Attitude (A): Attitude modification was addressed by creating a supportive environment where children could openly discuss their feelings and beliefs about PV [12]. Activities included role-playing, group discussions, and sharing personal experiences. These activities were designed to challenge negative attitudes and reinforce positive ones. Attitude changes were assessed using questionnaires that gauged shifts in participants’ beliefs and perceptions.

Changes in Practice (P): Behavioral changes were encouraged through practical skill-building exercises. Children were taught and practiced effective coping strategies, assertiveness training, and problem-solving skills [34]. These practices were reinforced through regular follow-up sessions. Behavioral changes were monitored through self-reports and observations by facilitators.

The intervention group received a 12-month intervention with a one-month gap between each session. The intervention consisted of educational presentations and videos delivered on-site, with each session lasting 30 to 40 min. The content of the intervention was shown in Table 1.

**Table 1:** Implementation of the PV intervention study for rural left-behind children from 2021 to 2022

Numbers	Time	Intervention theme	Purpose
1	2021.9	Types, damage and prevention of PV	Changing knowledge, practice and attitudes
2	2021.10	School bullying and school violence	Changing knowledge practice attitudes, subjective normative aspects
3	2021.11	Education about sexual safety	Changing knowledge practice attitudes, subjective normative aspects
4	2021.12	Prevention of sexual abuse	Changing knowledge, practice and attitudes
5	2022.1	Electronic victimization damage and prevention	Changing knowledge, practice and attitudes
6	2022.2	Emotional abuse and neglect	Changing knowledge, practice and attitudes
7	2022.3	Interpersonal communication among peers	Changing knowledge, practice and attitudes
8	2022.4	Non-violent communication, respect for empathy	Changing knowledge, practice and attitudes
9	2022.5	Case study discussion	Increased perceived practical control, shifted practical intentions
10	2022.6	Management of stress and emotions	Increased perceived practical control
11	2022.7	Reaction to stress and frustration	Increased perceived practical control
12	2022.8	Identification of psychological crises	Changing subjective normative aspects

### Control groups

The control group received general health education, which included information on preventing traffic accidents and drowning. To ensure the results were not confounded by extraneous variables, the length of the intervention and the interval between sessions were kept consistent with those of the intervention group.

### Ethics approval and consent to participate

All methods were performed in accordance with the relevant guidelines and regulations. This research was approved by the ethics committee of Shantou University Medical College. All the participants and their guardians agreed and provided signed, informed assent or consent on a voluntarily basis.

### Measurements

#### Victimization-related KAP scores

“Victimization-Related KAP Scores” is based on the theories developed by G. Cust [35], a British health educator, as well as the themes of this study on poly-victimization interventions. The questionnaire about victimization contained a total of 10 items for knowledge (K), 20 items for attitude (A), and 10 items for practice (P). The total KAP score was obtained by adding the attitude score, knowledge score, and practice score. For each item, the correct option was determined through prior discussion among experts, and the knowledge rate for each item was calculated for all children. The children’s KAP items on victimization were scored as follows: 1 point was given for each correctly answered item, and 0 points for each incorrectly answered item. The maximum score was 40 points. The overall reliability coefficient was 0.85, indicating high internal consistency. Each subscale also demonstrated acceptable reliability with coefficients above 0.75. Construct validity was assessed using factor analysis, which confirmed that the items loaded appropriately onto their respective factors (knowledge, attitude, and practice) with factor loadings above 0.70.

### Occurrence of poly-victimization

2 $$ \begin{aligned} & {\text{Victimization}}\;{\text{incidence}}\;{\text{rate}} \\ & \quad = {\text{number}}\;{\text{of}}\;{\text{victims}}\;{\text{per}} \\ & \quad \quad \;{\text{year}}/{\text{total}}\;{\text{number}}\;{\text{of}}\;{\text{people}}*100\% \\ \end{aligned} $$

3 $$ \begin{aligned} & {\text{Average}}\;{\text{number}}\;{\text{of}}\;{\text{victimization}}\;{\text{per}}\;{\text{year}} \\ & \quad = {\text{annual}}\;{\text{number}}\;{\text{of}}\;{\text{victimization}}\\ & \qquad \;{\text{incidents}}/{\text{number}}\;{\text{of}}\;{\text{victims}} \\ \end{aligned} $$

### Indicators for evaluating the effectiveness of interventions

4 $$ \begin{aligned} & {\text{Index}}\;{\text{of}}\;{\text{effectiveness}} \\ & = {\text{PV}}\;{\text{incidence}}\;{\text{rate}}\;{\text{of}}\;{\text{left}} \\ & \quad - {\text{behind}}\;{\text{children}}\;{\text{in}}\;{\text{the}}\;{\text{control}} \\ & \qquad \;{\text{group}}/{\text{PV}}\;{\text{incidence}}\;{\text{rate}}\;{\text{of}}\;{\text{left}} \\ & \quad - {\text{behind}}\;{\text{children}}\;{\text{in}}\;{\text{the}}\;{\text{intervention}}\;{\text{group}} \\ \end{aligned} $$

5 $$ \begin{aligned} & {\text{Protective}}\;{\text{rate}} \\ & = \left( {{\text{PV}}\;{\text{incidence}}\;{\text{rate}}\;{\text{of}}\;{\text{left}}} \right. \\ & \quad - {\text{behind}}\;{\text{children}}\;{\text{in}}\;{\text{the}}\;{\text{control}}\;{\text{group}} \\ & \quad - {\text{PV}}\;{\text{incidence}}\;{\text{rate}}\;{\text{of}}\;{\text{left}} \\ & \quad - {\text{behind}}\;{\text{children}}\;{\text{in}}\;{\text{the}}\;{\text{intervention}} \\ & \qquad \;\left. {{\text{group}}} \right)/{\text{PV incidence rate of left}} \\ & \quad - {\text{behind children in the control group}}*100\% \\ \end{aligned} $$

Index of effectiveness is calculated as the ratio of the PV incidence rate in the control group to the PV incidence rate in the intervention group. A higher index value indicates a more effective intervention, as it shows a greater relative reduction in PV incidence among the intervention group compared to the control. The effectiveness index is compared against a criterion of 1.0. An index value: Greater than 1.0 indicates that the intervention was effective in reducing PV rates compared to the control group. Equal to 1.0 suggests no difference in PV rates between the two groups. Less than 1.0 would imply that the intervention group had higher PV rates than the control group, suggesting ineffectiveness.

### Coping styles questionnaire

Coping styles were evaluated with the Coping Style Questionnaire (CSQ) [36], which is a 62-item self-report test. Items are rated as 1 (agree) or 0 (disagree). The questionnaire comprises six subscales including both immature and mature coping styles. Immature coping styles include “avoidance”, “fantasy” and “venting”; mature coping styles include “problem solving” “help seeking” and “bear”. In the original development of the CSQ, the construct validity was established with each factor consisting of entries with factor loadings greater than or equal to 0.35, and the overall reliability was recorded at 0.72. These metrics confirm the tool’s ability to reliably and validly measure coping behaviors. In the present study, the Cronbach alpha coefficient for the CSQ was 0.873, and the validity was 0.869, ensuring that the questionnaire remains a reliable and valid instrument for assessing coping styles in this study.

### Poly-victimization

Poly-victimization among children was examined by combining the JVQ-R2 (Juvenile Victimization Questionnaire-2nd Revision) developed by Finkelhor et al. [37] and the Chinese version of the JVQ [38]. The JVQ comprises six modules: Conventional crimes(9 items), Caregiver victimization(4 items), peer and sibling victimization(6 items), sexual victimization(6 items), Witnessing and indirect victimization(7 items), and Electronic victimization(2 items), encompassing 34 items in total. Each item relates to a specific type of harmful event, and subjects are asked to indicate whether such events have occurred. Scoring is binary; “yes” responses are valued at 1 point and “no” responses at 0 points. In the present sample, reliability and validity of the scale were tested; the standardized Cronbach’s alpha coefficient for the total score of recent victimization items is 0.875, with a KMO value of 0.793. According to prior studies, this study operationally defines Poly-victimization as a JVQ scale score of 4 points or more in the last year [39, 40].

### Data analysis

The study assesses the effectiveness of the intervention by comparing the KAP score and coping styles score differences before and after the intervention between the intervention group and the control group using Mixed Anova in SPSS 25.0. The count data were examined using a chi-square test to identify any variations in rates and composition ratios. The researchers utilized the Standardized Mean Difference (SMD) [41] metric to evaluate the equilibrium among the groups, with SMD < 0.1 signifying a satisfactory balance. This threshold indicates a minimal disparity between the groups under study. The expression was:

6 $$d = \frac{{\left| {p_{T} - p_{C} } \right|}}{{\sqrt {\frac{{p_{T} \left( {1 - p_{T} } \right) + p_{C} \left( {1 - p_{C} } \right)}}{2}} }}$$

In the above formula, *P*_*T*_ and *P*_*C*_ represent the positive rate of a covariate in the intervention group and the control group, respectively.

The efficacy of the intervention was assessed through the application of a double difference model, specifically the Difference-in-Differences (DID) method. This approach was utilized to analyze variations in Knowledge, Attitudes, and Practices (KAP), incidence of victimization, and coping mechanisms both pre- and post-intervention, as well as to determine the impact of the intervention within the intervention and control groups.

The DID model is constructed on the basis of the Ordinary least squares (OLS) method, and its generalized linear model expression for the double difference model is

7 $$Yit = \alpha _{{\text{0}}} + \alpha _{{\text{1}}} * Tit + \alpha _{2} * Dit + \alpha _{3} * Tit*Dit + \varepsilon it$$

The variable *T* serves as a categorical placeholder; when an individual (*i)* is impacted by the intervention, they are categorized into the intervention group denoted by *T* = 1. Conversely, if the individual (*i*)is not influenced by the intervention, they are categorized into the control group with *T* = 0. *D* is a dummy variable for the implementation of the intervention, where *D* = 0 before the intervention and *D* = 1 after the intervention. Tit * Dit is the interaction term between the grouping dummy variable and the intervention implementation dummy variable with coefficient *α*_*3*_.

## Results

### Baseline characterization

The baseline characteristics of the control and intervention groups are presented in Table 2. Each group contained 228 subjects. The differences in the distribution proportions of gender, only-child status, health status, unscrupulous behavior of close friends, parental marital status, and parental health status did not exhibit statistical significance between the two groups (*p*>0.05). However, the distribution proportions of parental occupation demonstrated statistical significance (*p*<0.05). Gender, only-child status, health status, parental marital status, and parental health status had SMD < 0.1, while unscrupulous behavior of close friends and parental occupation had SMD > 0.1.

**Table 2:** Baseline characteristics of the Control and Intervention Groups among left-behind children

	The control group (*n* = 228)	The intervention groups (*n* = 228)	*p*	SMD
Age	13.28 ± 0.232	13.66 ± 0.342	0.621	
Grade				
First grade	122 (53.51)	113 (49.56)	0.399	0.034
Second grade	106 (46.49)	115 (50.44)		
Gender			0.779	0.026
Females	119 (52.19)	116 (50.88)		
Males	109 (47.81)	112 (49.12)		
Only-child status			0.482	0.066
No	217 (95.18)	220 (96.50)		
Yes	11 (4.82)	8 (3.51)		
Health status			0.503	0.063
Healthy	219 (96.05)	216 (94.74)		
Disabled	9 (3.95)	12 (5.26)		
Unscrupulous behavior of close friends			0.16	0.132
No	204 (89.47)	194 (85.09)		
Yes	24 (10.53)	34 (14.91)		
Parental marital status			0.878	0.014
Marriage	205 (89.91)	204 (89.47)		
Divorced/Widowed	23 (10.09)	24 (10.53)		
Father’s occupation			0.002	0.218
White collar	42 (18.42)	18 (7.89)		
Blue collar	175 (76.75)	203 (89.04)		
Unemployed	11 (4.83)	7 (3.07)		
Mother’s occupation			0.046	0.128
White collar	32 (14.04)	16 (7.02)		
Blue collar	149 (65.35)	165 (72.37)		
Unemployed	47 (20.61)	47 (20.61)		
Parents’ health status			0.16	0.041
Healthy	218 (95.61)	216 (94.74)		
Disabled	10 (4.39)	12 (5.26)		

Percentages are in parentheses, mean ± standard deviation

### Variations in KAP related to victimization before and after intervention

A 2 × 2 mixed ANOVA was conducted with group (intervention and control) as the between subjects factor and time (before and after intervention) as the within subjects factor. The results showed a significant main effect for intervention, F(1, 454) = 25.905, *p* < 0.05. For group, control group reported significantly less KAP score than intervention group. There was also a significant group × time interaction, F(1, 454) = 42.142, *p* < 0.05. The results of the simple effects tests indicated that control group reported significantly less KAP score than intervention group before intervention, ( *p* < 0.05). The mean and standard deviation of the KAP and its subcategory scores are reported in the following table (Tables 3, 4).

**Table 3:** KAP scores before and after the intervention in the intervention and control groups of left-behind children

Variables	Time	Intervention group		Control group	
		M	SD	M	SD
Attitude score	Before intervention	15.15	3.23	15.81	3.24
Attitude score	After intervention	16.89	2.84	15.90	3.02
Knowledge score	Before intervention	5.22	4.17	5.51	3.93
Knowledge score	After intervention	6.62	3.50	5.28	4.07
Practice score	Before intervention	4.89	4.00	5.34	3.86
Practice score	After intervention	6.54	3.43	5.04	3.96
KAP score	Before intervention	25.26	9.96	26.66	9.07
KAP score	After intervention	30.05	8.42	26.22	9.36

**Table 4:** DID model of KAP scores for left-behind children

	DID	S. E.	t	*p*
Attitude score	1.732	0.409	4.24	0.000^***^
Knowledge score	1.864	0.518	3.6	0.000^***^
Practice score	2.171	0.504	4.31	0.000^***^
KAP score	5.768	1.217	4.74	0.000^***^
KAP awareness rate	0.144	0.03	4.74	0.000^***^

^***^<0.001

### Variations in PV occurrence before and after the intervention

During the initial survey, the prevalence of PV among children in the intervention group was 20.2% (*n* = 46), while in the control group it was 25.4% (*n* = 58), indicating no statistically significant difference (*p* = 0.18). The incidence of PV in the control group was 25.4% during the initial survey and increased to 36.0% (*n* = 82) following the intervention, demonstrating a statistically significant difference (*p* = 0.10). Following the intervention, the prevalence of PV among children in the intervention group declined to 9.6% (*n* = 22), demonstrating a statistically significant decrease compared to the baseline survey findings (*p* < 0.01). The prevalence of PV among children in the intervention group was found to be significantly lower compared to the control group, with statistical significance (*p* < 0.01). The net reduction in the incidence of PV among children was 21.2%. The post-intervention protection against the occurrence of PV among children was 73.33% with an effectiveness index of 3.75 (Table 5).

**Table 5:** Variations in the number of different types of victimization occurring before and after the intervention for left behind children

Number	Intervention group (*n* = 228)		*p*	Control group (*n* = 228)		*p*	Difference (%)		Net difference (%)	Protective rate (%)	Effectiveness index
	Before intervention	After intervention		Before intervention	After intervention		Intervention group	Control group			
0	119 (52.19)	155 (67.98)	0.001	104 (45.61)	88 (38.60)	0.197	15.80	-7.00	22.80		
1	38 (16.67)	26 (11.40)		33 (14.47)	30 (13.16)		− 5.30	− 1.30	− 4.00	13.37	1.16
2	13 (5.70)	13 (5.70)		19 (8.33)	16 (7.02)		0.00	− 1.30	1.30	18.80	1.23
3	12 (5.26)	12 (5.26)		14 (6.14)	12 (5.26)		0.00	− 0.80	0.80	0.00	1.00
≥ 4 (PV)	46 (20.18)	22 (9.66)		58 (25.45)	82 (35.96)		− 10.60	10.60	− 21.20	73.14	3.75

The changes in the distribution of victimization types before and after the intervention are presented in Table 6. The incidence of various types of victimization changed differently following the intervention. The predominant types of victimization observed in children before and after the intervention primarily consisted of conventional crimes and witnessing and indirect victimization. The most effective interventions were observed in cases of peer victimization and electronic victimization, where protection rates exceeded 60% and the effectiveness indices were recorded at 2.80 and 2.81, respectively.

**Table 6:** Variations in the occurrence of different types of victimization before and after the intervention in the intervention and control groups of left behind children

	Control group		*p*	Intervention group		*p*	Difference (%)		Net difference (%)	Protection rate (%)	Effect Index
	Before intervention	After intervention		Before intervention	After intervention		Control group	Intervention group			
Conventional crimes			0.038			0.00					
No	140 (61.40)	118 (51.80)		155 (68.00)	188 (82.80)						
Yes	88 (38.60)	110 (48.20)		73 (32.00)	39 (17.20)		9.60	− 14.80	− 24.40	64.32	2.80
Child abuse			0.197			0.00					
No	188 (82.50)	177 (77.60)		188 (82.50)	198 (86.84)						
Yes	40 (17.50)	51 (22.40)		40 (17.50)	30 (13.10)		4.90	− 4.40	− 9.30	41.07	1.70
Peer victimization			0.156			0.03					
No	179 (78.50)	166 (72.80)		177 (77.60)	193 (97.80)						
Yes	49 (21.50)	62 (27.20)		51 (22.40)	35 (15.35)		5.70	− 7.05	− 12.75	43.56	1.77
Electronic victimization			0.342			0.023					
No	188 (82.50)	180 (78.90)		196 (86.00)	211 (92.50)						
Yes	40 (17.50)	48 (21.10)		32 (14.00)	17 (7.50)		3.60	− 6.50	− 10.10	64.45	2.81
Sexual victimization			0.435			0.208					
No	216 (94.70)	212 (93.00)		213 (93.40)	219 (96.10)						
Yes	12 (5.30)	16 (7.00)		15 (6.60)	9 (3.90)		1.70	− 2.70	− 4.40	44.29	1.79
Witnessing and indirect victimization			0.059			0.32					
No	162 (71.10)	143 (62.70)		185 (81.10)	193 (84.60)						
Yes	66 (28.90)	85 (37.30)		43 (18.90)	35 (15.40)		8.40	− 3.50	− 11.90	58.71	2.42

Subgroup dummy variables (control group d = 0; intervention group d = 1), time dummy variables (after intervention t = 1; before intervention t = 0), and interaction terms between subgroups and time (td) were set according to the requirements of the DID model. The data presented in Table 7 demonstrate that the intervention resulted in a statistically significant decrease in the occurrence of victimization among children.

**Table 7:** The effect of intervention on the number of victimization in left-behind children

Variable	Victimization number	S. E.	t	*p*
Before intervention				
Control group	2.272			
Intervention group	1.864			
Difference(intervention-control)	− 0.408	0.294	− 1.39	0.166
After intervention				
Control group	3.075			
Intervention group	0.982			
Difference(intervention-control)	− 2.092	0.287	7.29	0.000^***^
DID	− 1.684	0.411	4.1	0.000^***^

^***^<0.001

### Variations in coping styles before and after the intervention

A 2 × 2 mixed ANOVA was conducted with group (intervention and control) as the between subjects factor and time (before and after intervention) as the within subjects factor. The results showed a significant main effect for intervention, F(1, 454) = 9.554, *p* < 0.05. For group, control group reported significantly less total coping styles score than intervention group. There was also a significant group × time interaction, F(1, 454) = 20.004, *p* < 0.05. The results of the simple effects tests indicated that control group reported significantly less coping styles score than intervention group before intervention, ( *p* < 0.05). The mean and standard deviation of the coping styles scores and its subcategory scores are reported in the following table (Table 8).

Dummy variables were created to represent subgroups (control group d = 0 and intervention group d = 1), time points (before intervention t = 0 and after intervention t = 1), and the interactions between subgroups and time (td), as specified by the DID model. The intervention led to an increase in children’s competence in positive coping styles when facing victimization, including problem-solving and help-seeking, while concurrently reducing competence in negative coping styles, such as avoidance and venting. The observed difference was statistically significant (*p* < 0.05) (Table 9).

**Table 8:** Differences in the occurrence of different coping styles before and after intervention for left-behind children note: Mean ± standard deviation

Dimensions of coping styles	Control group		Intervention group	
	Before	After	Before	After
Bear	2.92 ± 0.94	2.65 ± 0.81	2.85 ± 0.87	2.58 ± 0.91
Fantasy	1.98 ± 0.97	2.23 ± 0.80	2.00 ± 0.89	2.19 ± 0.89
Venting	2.28 ± 0.815	2.35 ± 0.71	2.38 ± 0.64	2.05 ± 0.73
Avoidance	2.70 ± 0.80	2.61 ± 0.68	2.76 ± 0.75	2.43 ± 0.72
Help-seeking	2.59 ± 0.81	2.57 ± 0.71	2.39 ± 0.75	2.705 ± 0.82
Problem solving	2.99 ± 0.78	2.77 ± 0.71	3.05 ± 0.80	4.92 ± 1.39
Total scores	15.47 ± 3.73	15.20 ± 3.49	15.43 ± 2.92	16.92 ± 3.37

**Table 9:** A DID model of intervention effects on coping styles of left-behind children

Coping styles	DID	S. E.	t	*p*
Problem solving	2.142	0.128	16.79	0.000^***^
Help-seeking	0.343	0.103	3.33	0.001^***^
Avoidance	-0.242	0.099	2.46	0.014^*^
Venting	-0.393	0.097	4.06	0.000^***^
Fantasy	-0.05	0.121	0.41	0.679
Bear	0.004	0.118	0.04	0.972

^*^p < 0.05,^**^p < 0.01,^***^p < 0.001

## Discussion

The primary purpose of this study was to evaluate the effectiveness of an intervention program based on the Theory of Planned Behavior in reducing poly-victimization among left-behind children in rural China. Additionally, we aimed to assess the impact of the intervention on the Knowledge, Attitudes, and Practice (KAP) related to victimization and the coping styles of the participants. The results of the study showed that after the intervention, the incidence of PV in left-behind children decreased by 21.20%. Therefore, the intervention implemented in this study may have been effective in reducing the incidence of PV in left-behind children. After the intervention, the KAP scores related to victimization among left-behind children were significantly improved compared to the baseline survey. Therefore, such interventions can lead to enhanced levels of KAP related to PV among children. The results of this study showed that the intervention increased children’s competence in positive coping styles when facing victimization, such as problem-solving and help-seeking, while decreasing competence in negative coping styles, such as avoidance and venting, with statistically significant differences (*p* <0.05). This suggests that interventions based on the TPB framework can improve the coping styles of left-behind children and thus reduce the occurrence of PV.

Our findings align with existing literature that underscores the effectiveness of interventions based on cognitive-behavioral frameworks like TPB in changing behavior and improving psychological outcomes [31]. Research has shown that structured interventions can significantly impact knowledge and attitudes towards complex issues like victimization [42]. Furthermore, the improvement in coping strategies observed in our study reflects findings from prior studies, which indicate that positive coping styles can mitigate the impacts of adverse experiences [43]. The reduction in PV rates and improvement in KAP scores in our study are consistent with the theoretical predictions of TPB, which suggest that changes in attitudes, subjective norms, and perceived behavioral control can lead to behavior change. The enhancement of coping strategies supports literature emphasizing the role of adaptive coping in reducing the occurrence and impact of negative life events [44].

Prior studies have indicated that experiences of PV can impact the coping styles of children and adolescents when faced with challenges. Coping refers to the cognitive and practical strategies employed by an individual in response to stressful situations to reduce or alleviate the negative impacts of stress [45]. Academics widely recognize coping as a dynamic combination of cognition and behavior. Coping styles may also impact the occurrence of PV, with positive coping styles mitigating the negative effects of adverse events [46, 47]. Some studies have also suggested that the more frequently negative coping styles are used, the higher the frequency of adverse life events. Appropriate coping styles may not only reduce negative affect but also reduce the frequency of individual injurious events and the number of types of victimization, thereby preventing the occurrence of PV [48–50].

The study employed a rigorous difference-in-differences (DID) analysis to evaluate the intervention’s effectiveness, providing robust causal inference. The intervention was based on the well-established Theory of Planned Behavior, which has been shown to be effective in various health-related behavior change interventions. This robust statistical approach allows us to confidently attribute observed changes in poly-victimization and coping strategies directly to the intervention. This analytical rigor is complemented by the theoretical foundation of the intervention, which is based on the well-established Theory of Planned Behavior (TPB). The study included a sizable sample of left-behind children from multiple schools, focusing on this particular group. Additionally, the focus on left-behind children, a particularly vulnerable population, not only enhances the generalizability of our findings within this demographic but also addresses a significant gap in the literature. By integrating a sizable sample from multiple schools, the study aims to mitigate challenges faced by these children. However, this study is not without several limitations. First, Some of the study data relied on self-reported measures, which may be subject to social desirability bias and recall bias. Second, the follow-up period lasted for a relatively short period of about one year, limiting the assessment of the long-term sustainability of the intervention effects. Third, the study did not compare the intervention group with a group receiving a different type of intervention, such as social-emotional learning, which could have provided a more comprehensive understanding of the relative effectiveness of different intervention strategies. Fourth, some participants in the intervention group transitioned from PV to non-PV, while others moved from non-PV to PV status. However, our study did not focus on those who transitioned from non-PV status (having experienced 0, 1, 2, or 3 victimization incidents) to PV status (experiencing 4 or more incidents), nor on the differences in protection rates among groups subjected to varying numbers of victimization. Future studies could conduct long-term follow-up studies to assess the sustainability of the intervention effects, or explore the potential mediating and moderating factors that influence the effectiveness of interventions in reducing PV among left-behind children, to promote the healthy growth of the special group of left-behind children.

## Conclusion

Interventions based on the Theory of Planned Behavior reduced the incidence of poly-victimization in left-behind children, with effects varying across different types of victimization. The implementation of an intervention program based on the TPB should consider the prevalence characteristics of poly-victimization among left-behind children in the region and the influencing factors obtained from the baseline survey, and then formulate evidence-based, rational, and comprehensive interventions. Therefore, by adopting measures that consider the types of victimization in the intervention program, the occurrence of the main types of victimization can be effectively reduced.

## Data Availability

No datasets were generated or analysed during the current study.
